# Adult Clavicular Fracture Case Report

**DOI:** 10.21980/J8FM0T

**Published:** 2020-10-15

**Authors:** Jessica L Sea, Nadia Zuabi, Alisa Wray

**Affiliations:** * University of California, Irvine, Department of Emergency Medicine, Orange, CA

## Abstract

Clavicular fractures make up 2–4% of all fractures. While most are nondisplaced and easily managed, serious complications can arise from more severe fractures. In these cases, immediate surgical intervention is required. However, there lacks a consensus regarding the optimal treatment for fractures that are less severe. Here, we present a case of a 25-year-old male with a comminuted displaced mid-clavicular fracture. This report details the important clinical features of various types of clavicular fractures and discusses the current literature regarding indications for operative and nonoperative management.

**Topics:**

Adult clavicular fracture; mid-clavicle fracture; orthopedics, trauma, upper extremity.

**Figure:**
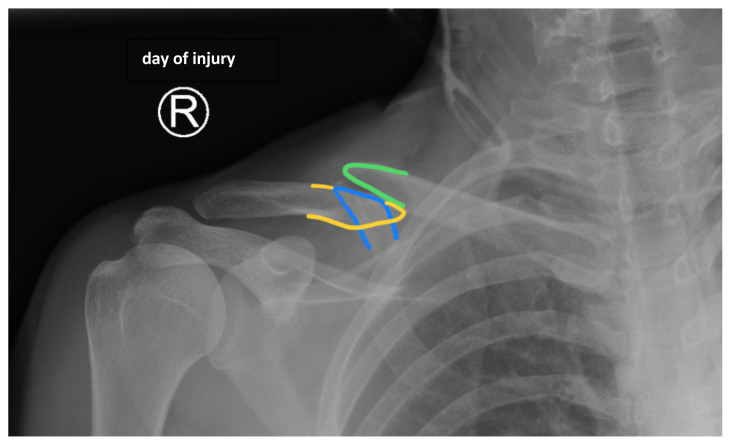

**Figure:**
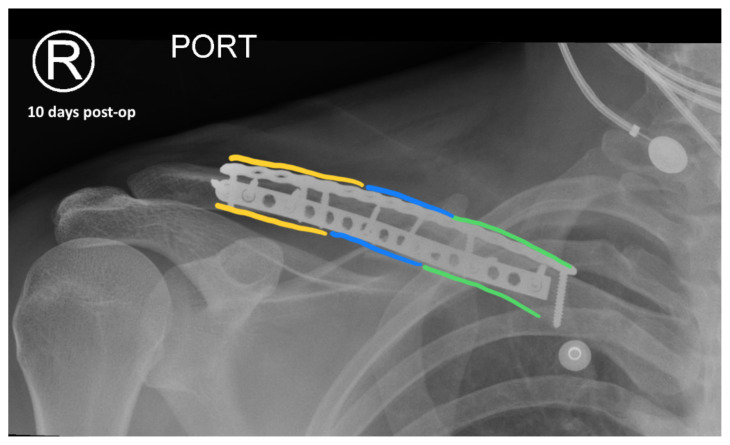

**Figure:**
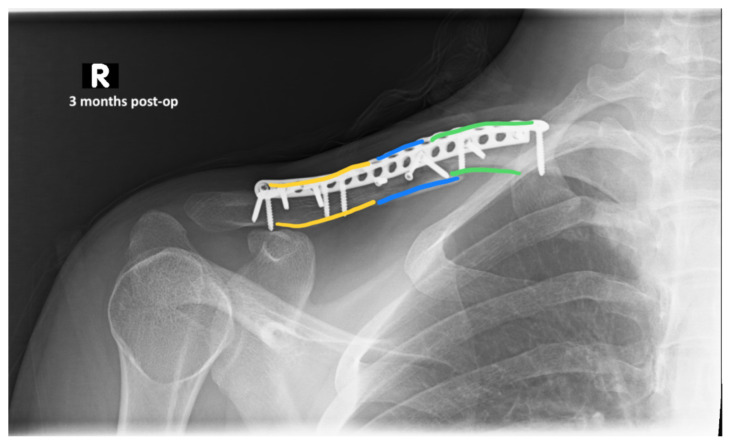

**Figure:**
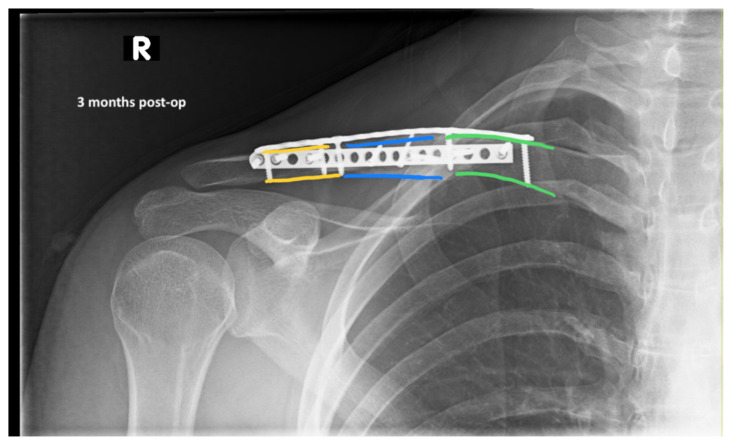

**Figure:**
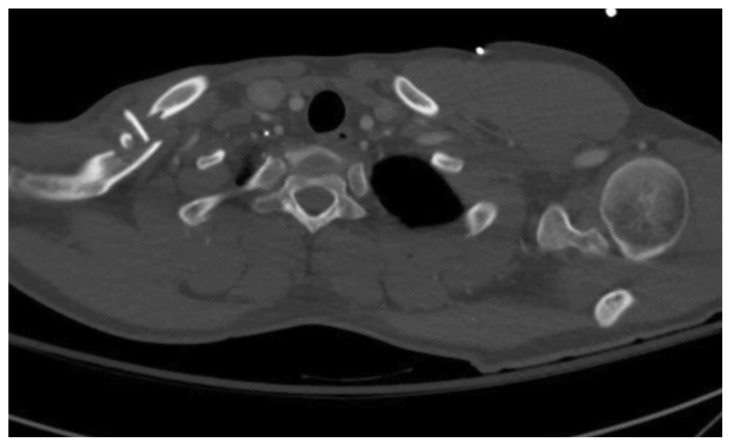
Video Link: https://youtu.be/hdkrN1KRIT4

## Presenting concerns and clinical findings

A 25-year-old male with no past medical history presented to the emergency department as a trauma activation with a chief concern of right clavicular pain after a motorcycle collision. The patient was well-appearing with normal vital signs and a Glasgow Coma Scale of 15. The patient received a full trauma evaluation by the emergency department and trauma teams. Physical examination revealed only right clavicle deformity and tenderness to palpation of his right clavicle and right upper chest wall. The patient was neurovascularly intact with good peripheral pulses, sensation, and full strength in his right upper extremity. He had significant pain with range of motion of his shoulder. The remainder of his examination was unremarkable, his vitals remained stable, and his bedside hemoglobin was in the normal range. Given the speed and mechanism of injury, the patient underwent computed tomography of the head, neck, chest, abdomen and pelvis, as well as plain radiographs of the chest, right clavicle and shoulder.

## Significant findings

The patient’s chest and clavicular radiographs showed a comminuted displaced acute fracture of the right mid-clavicle (green, blue, yellow). The clavicular fracture was also visible on the chest computed tomography (CT). The remainder of his trauma workup was negative for acute findings.

## Patient Course

The patient was cleared by trauma and admitted to the orthopedic service for surgical management of his clavicular fracture. On hospital day two, he was taken to the operating room for plating of his clavicular fracture given that it was comminuted and displaced. He was discharged postoperatively. Radiographic images obtained during the patient’s follow-up appointments showed typical healing patterns at 10 days (10 days post-operative, green, blue, yellow) and at three months post-operation (3 months post-operative, green, blue, yellow).

## Discussion

Clavicular fractures commonly present with point tenderness over the fracture accompanied by a visible bulge or deformity resulting from swelling, hematoma/ecchymosis, angulation, and/or displaced bone fragments. Associated pain is often well-localized and exacerbated by arm or shoulder movement. In most cases, clavicular fractures are confirmed by radiography, though fractures in the distal and proximal clavicle sometimes require CT imaging due to the nature and location of these injuries.^1^ Distal fractures can be mistaken for acromioclavicular (AC) separations and range in severity depending on the extent of damage to the surrounding ligaments and displacement of bone fragments. The most common clavicular fractures are those of the mid-clavicle, where significant bone displacement, shortening, and comminution are frequently noted.^1^,^2^ Though serious complications from clavicular fractures are rare, patients who have sustained high-impact trauma should be evaluated for emergent injuries. This is especially true of acute fractures in the proximal clavicle because nearly ninety percent are associated with multiorgan trauma.^1^ Neurovascular, lung, and cardiac exams are of particular importance in order to rule out concurrent hemothorax, pneumothorax, laceration of subclavian vessels, brachial plexus compression, and additional fractures to the scapula or ribs.

Management of clavicular fractures may involve either surgical or nonsurgical immobilization techniques depending on the location and severity of the fracture, and precipitating complications. Indications for immediate operative repair include open fractures, skin tenting, and injuries resulting in neurovascular, respiratory, or hemodynamic compromise.^3^

Fractures adjacent to an open wound should be considered open because bone can puncture through the tissue and recede back inside obscuring the bone from view. Skin tenting, where skin is pulled taut over the fracture, is often suggestive of significant angulation or displacement of the broken bone which also requires immediate attention. Fractures assessed in the emergency department may present with hemodynamic instability, indicative of intrathoracic hemorrhage or other serious complications requiring emergency surgery. Similarly, complications resulting in diminished respiratory function or neurovascular involvement also warrant immediate surgical intervention. While these situations clearly define the need for operative management, there remains some debate over the most optimal course of treatment for fractures lacking such complications.^4^,^5^

Nonoperative conservative treatment consists of fracture immobilization (typically with a sling, collar with cuff, or figure-eight bandage), pain management, and mobility exercises to prevent stiffening of the elbow and shoulder joints. It should also include a recommendation for patients to apply a cold compress for 20–30 minutes several times per day for the first 3 days after injury to ease pain and swelling. Pain can be managed initially with moderate strength opioids followed by acetaminophen after 3–7 days, or acetaminophen alone. Though the sling and figure-eight bandage are equally effective at immobilizing the injured area in most fractures, a few studies report higher patient satisfaction, improved ease of use, and reduced pain in the first few days after injury with a sling.^6^–^8^

Nondisplaced mid-clavicular fractures are most frequently treated conservatively due to the low incidence of nonunion.^9^ However, operative management is an important consideration for patients with physically demanding jobs or those who are particularly athletic since surgical repair has been associated with reduced clinical healing time, as well as patients with cosmetic concerns.^7^,^10^ There is conflicting evidence over whether surgical or conservative treatment is more beneficial; therefore, the best course of action is likely to be determined on a case by case basis. Operative treatment is recommended for patients who have mid-clavicular fractures with complete displacement, shortening greater than 2cm or 10% in length, or comminution. Surgery in these situations is associated with lower rates of malunion and nonunion, improved range of motion, and decreased cosmetic abnormalities. 5,7,11–13 Of note, while the degree of angulation is important within the context of complete displacement, shortening, and comminution, angulation alone is not regularly used to decide between conservative and operative treatment options. Lastly, midclavicular fractures resulting in a “floating shoulder,” in which there are ipsilateral clavicle and glenoid neck fractures, should be treated as urgent and referred to orthopedic surgery.

Distal clavicular fractures are divided into three groups. The most common of these is Type I which generally lacks displacement because ligaments remain intact, maintaining alignment of the distal bone fragment. Conservative management is recommended for type I due to the minimal displacement observed in these fractures.^14^ In type II, the proximal bone fragment becomes detached from the ligament and is displaced superiorly. These fractures are more prone to injury and require orthopedic surgery in most cases.^15^,^16^ Patients who decline surgery should be directed to use a sling in order to adequately support the weight of the arm and prevent further displacement. Similar to type I, type III also presents as an intraarticular fracture with intact ligaments that prevent displacement. Most type III fractures can be managed without surgery, although some patients may need to be referred to orthopedic surgery for evaluation since degenerative changes in the AC joint have been associated with type III distal fractures.^15^,^16^

Patients with acute proximal clavicular fractures require immediate evaluation for trauma-related injuries. Proximal clavicular fractures resulting from trauma are often associated with other serious injuries including hemothorax, pneumothorax, and hemopneumothorax. One study of 55 patients with proximal clavicular fractures reported that over half (55%) also presented with pulmonary contusions, respiratory failure, or adult respiratory distress syndrome.^1^ Patients can also present with spinal injury, rib fractures, and serious head injuries including subarachnoid hemorrhage. Alternatively, proximal fractures can also be caused by repetitive stress fractures. These are nondisplaced fractures that can be treated conservatively with a sling, ice, pain management, and if appropriate, temporary limitation of repetitive physical activities. A fracture resulting in the posterior displacement of a fragment or posterior sternoclavicular dislocation should be referred urgently to orthopedic surgery.

Of the different types of clavicular fractures that range in severity and incidence, many can be managed conservatively without the need for surgery. When possible, conservative treatment can be particularly beneficial for patients with less severe fractures who are not optimal candidates for surgery due to preexisting health conditions, age, or other factors. There are several elements to consider when deciding between immobilization and surgery that include potential complications, risks of nonunion or malunion, and the patient’s level of physical activity. It is recommended that physicians and patients discuss the potential risks and benefits associated with each treatment with regards to both short-term and long-term outcomes in order to determine the best course of action for that individual.

## Supplementary Information






















