# Supplementary figures and images for: Adult Clavicular Fracture Case Report
Source: J Educ Teach Emerg Med. 2020 Oct 15;5(4):V6–V11. doi: 10.21980/J8FM0T (PMC10332523; doi:10.21980/J8FM0T)

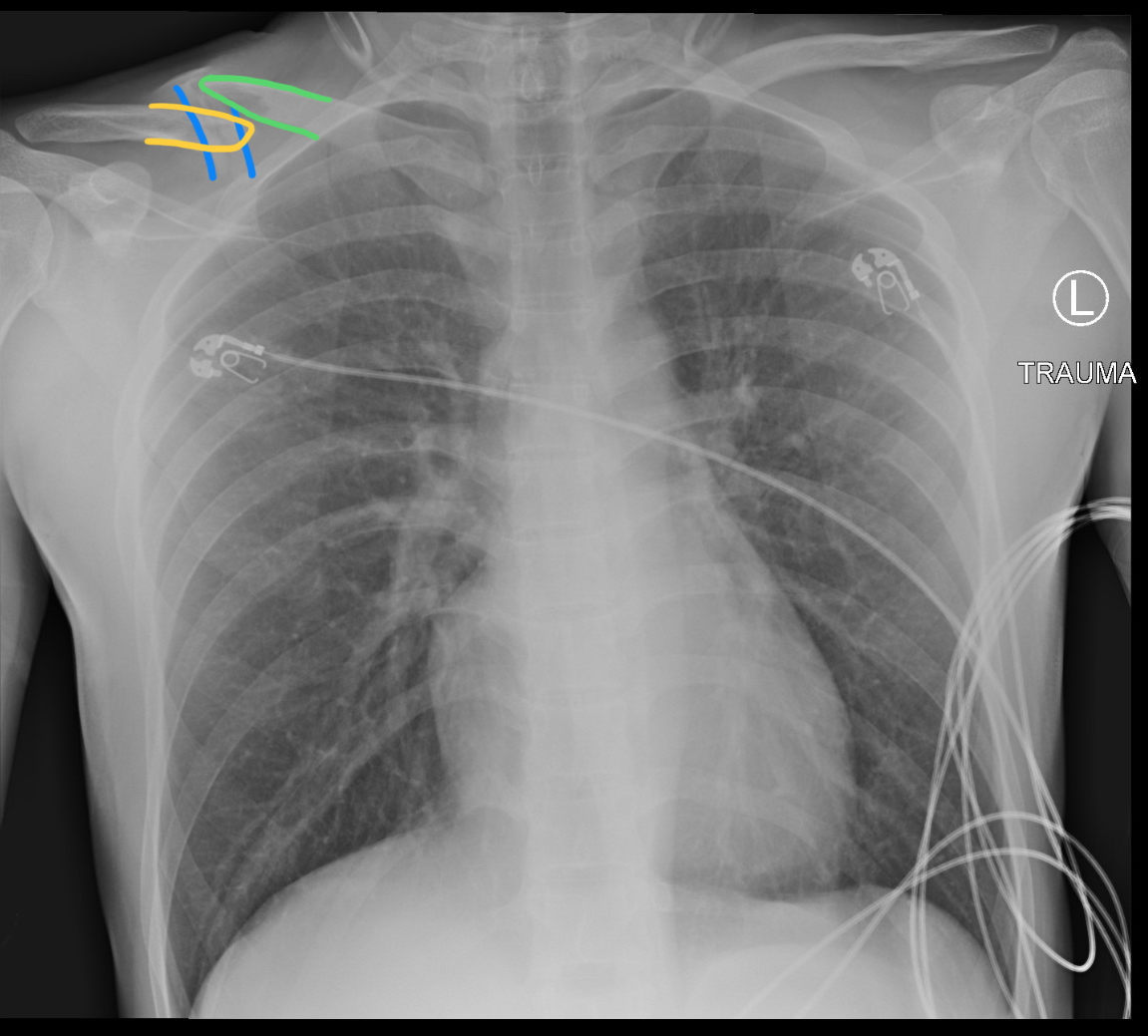

Supplement: Supplementary file 1 [file jetem-5-4-v6-supp1.jpeg]

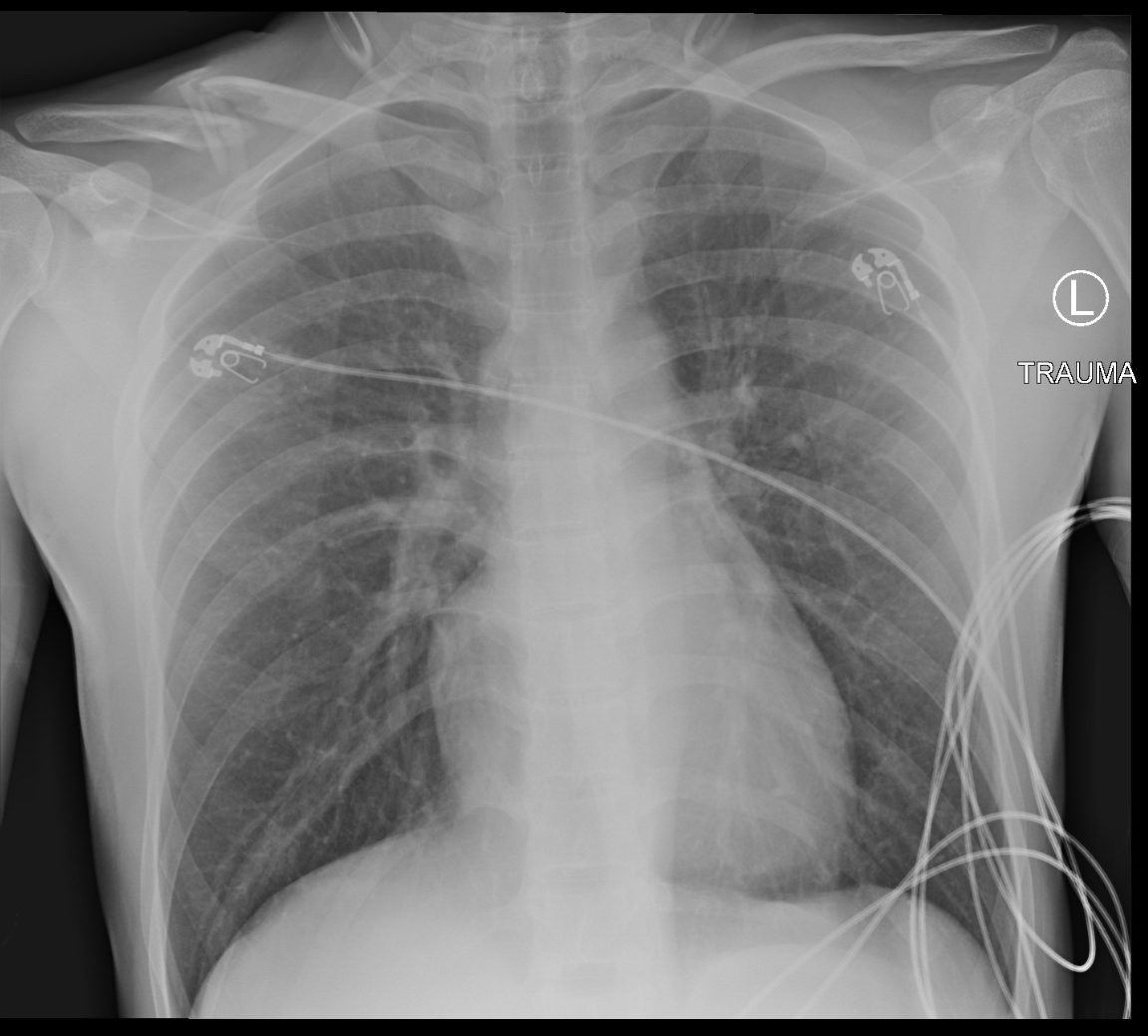

Supplement: Supplementary file 2 [file jetem-5-4-v6-supp2.jpeg]

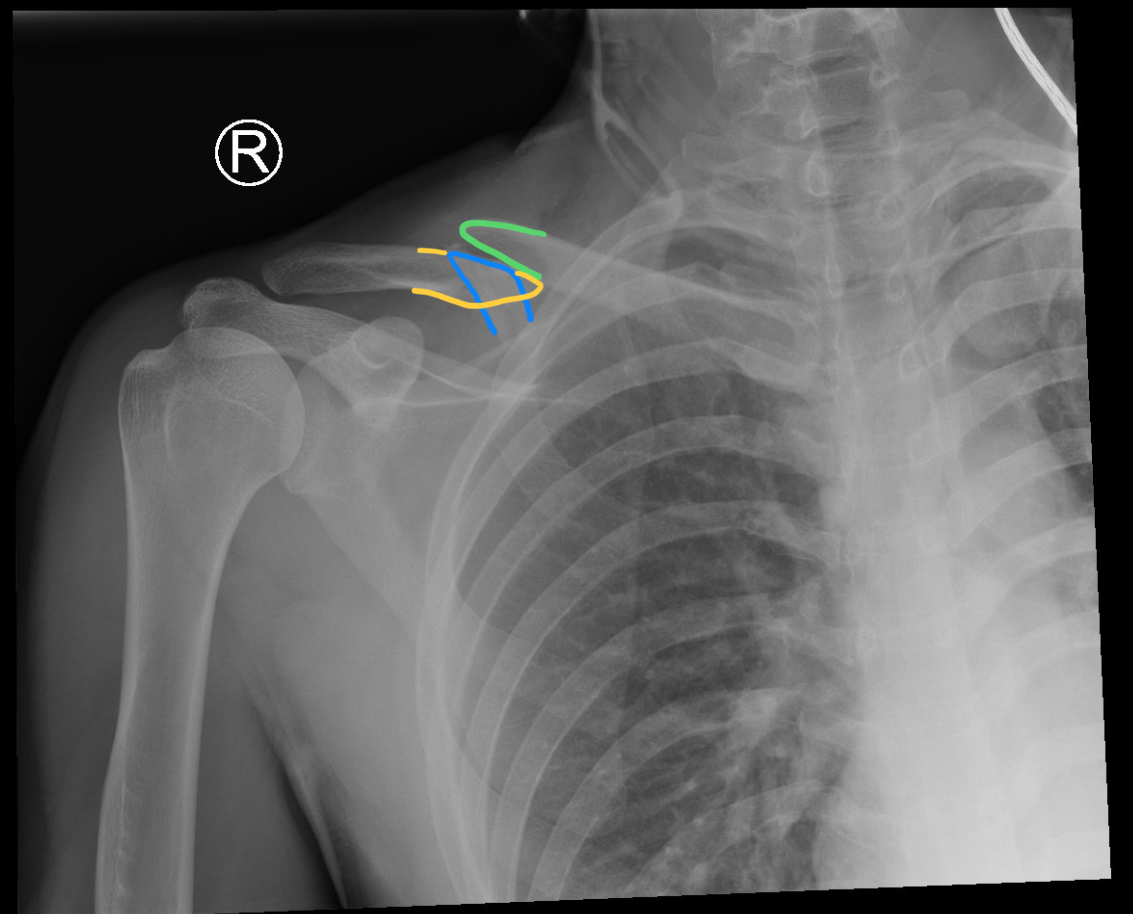

Supplement: Supplementary file 3 [file jetem-5-4-v6-supp3.jpeg]

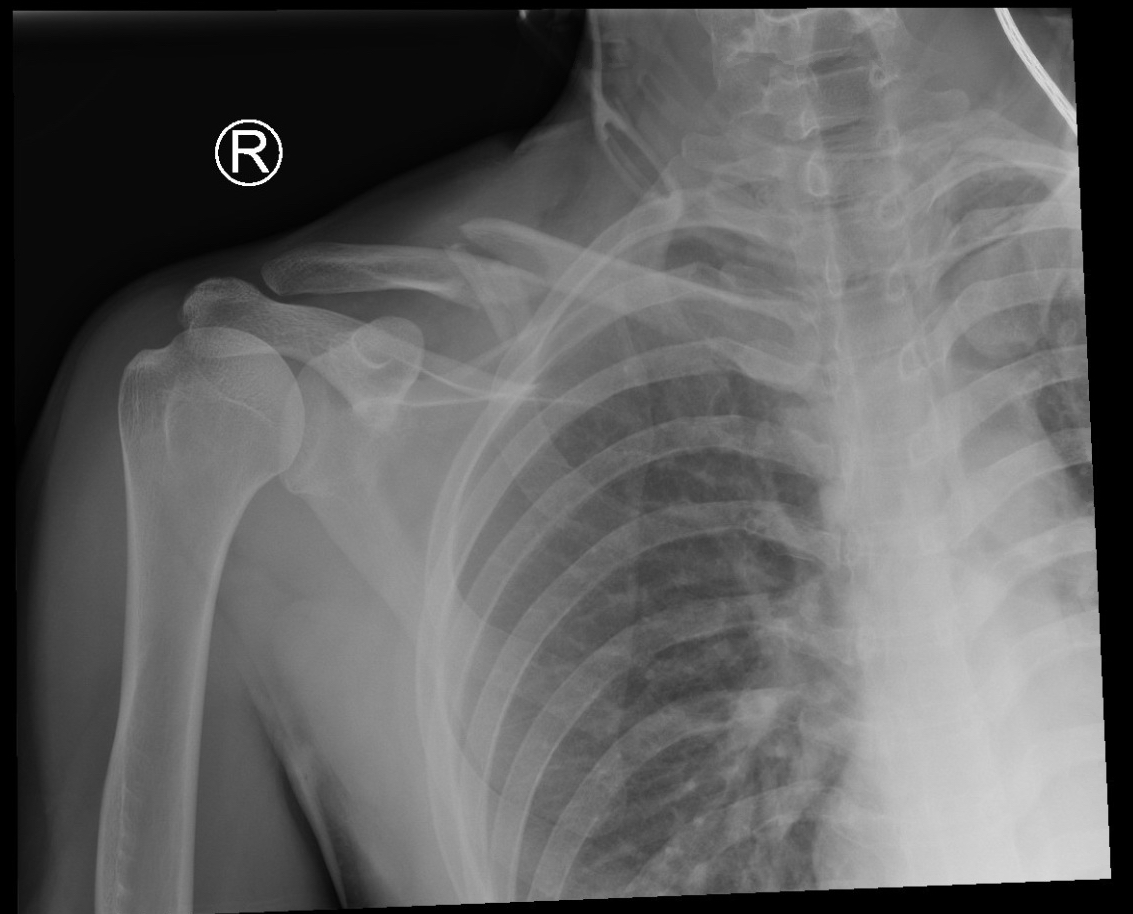

Supplement: Supplementary file 4 [file jetem-5-4-v6-supp4.jpeg]

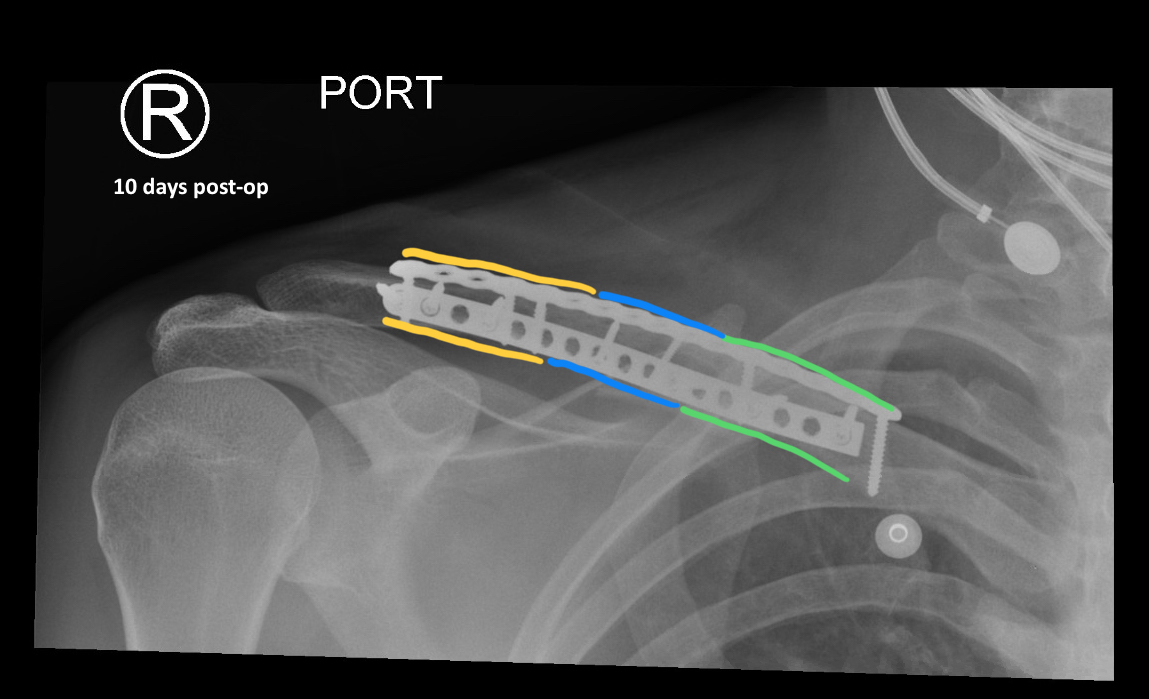

Supplement: Supplementary file 5 [file jetem-5-4-v6-supp5.jpeg]

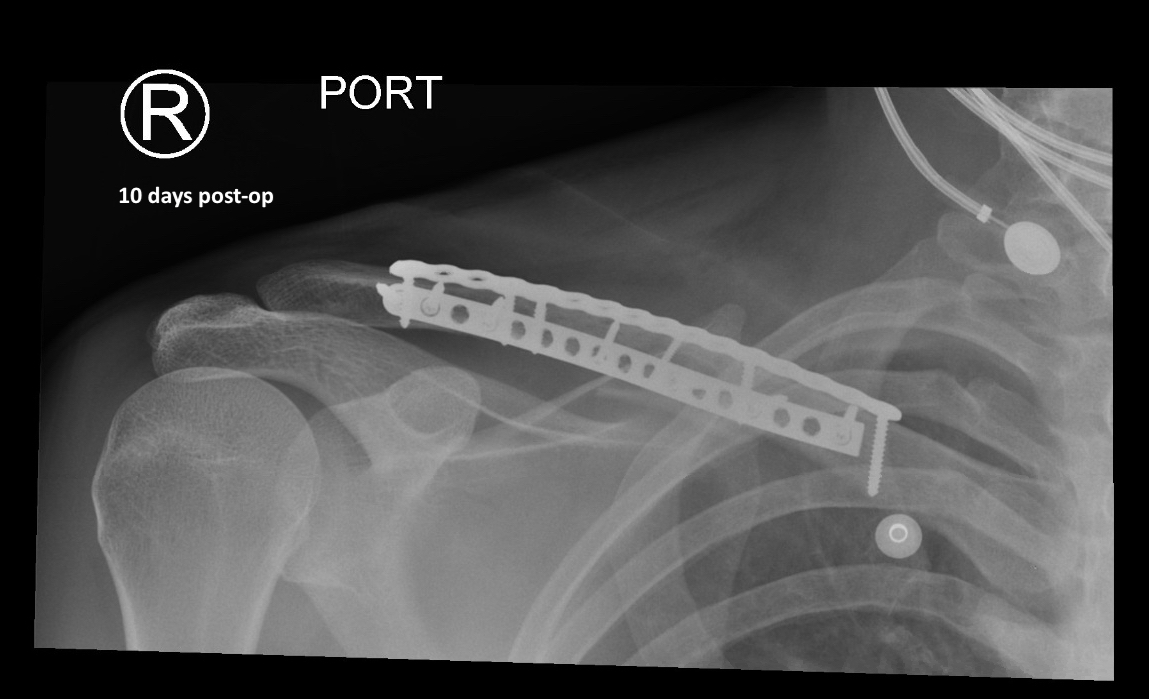

Supplement: Supplementary file 6 [file jetem-5-4-v6-supp6.jpeg]

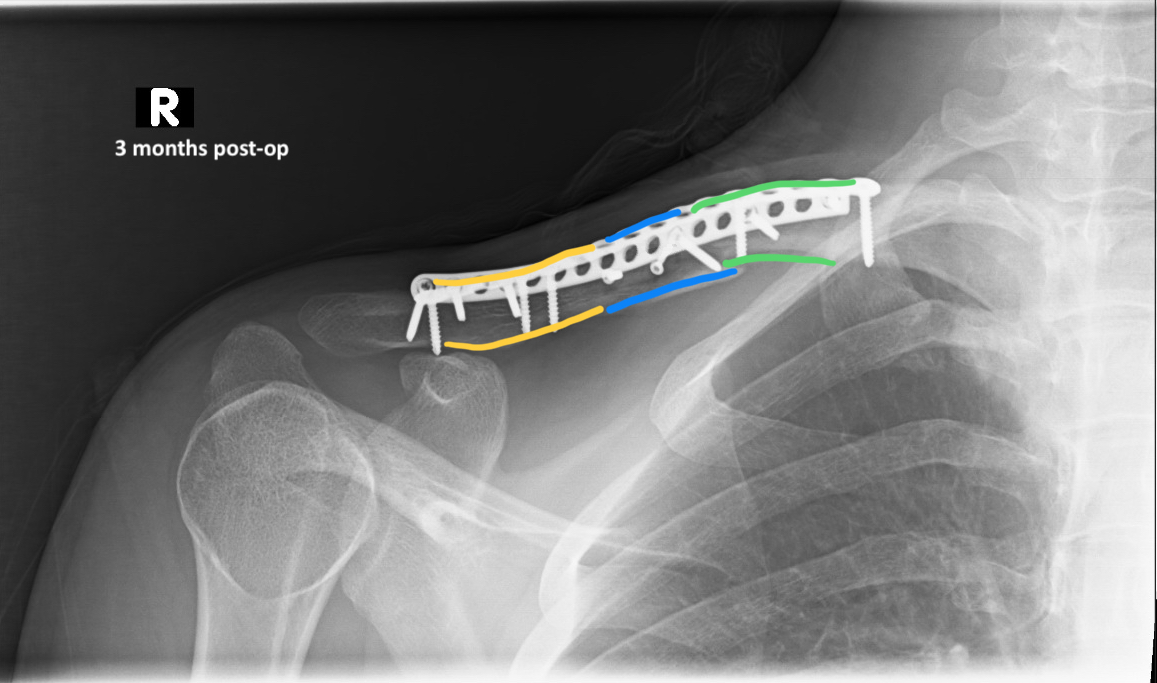

Supplement: Supplementary file 7 [file jetem-5-4-v6-supp7.jpeg]

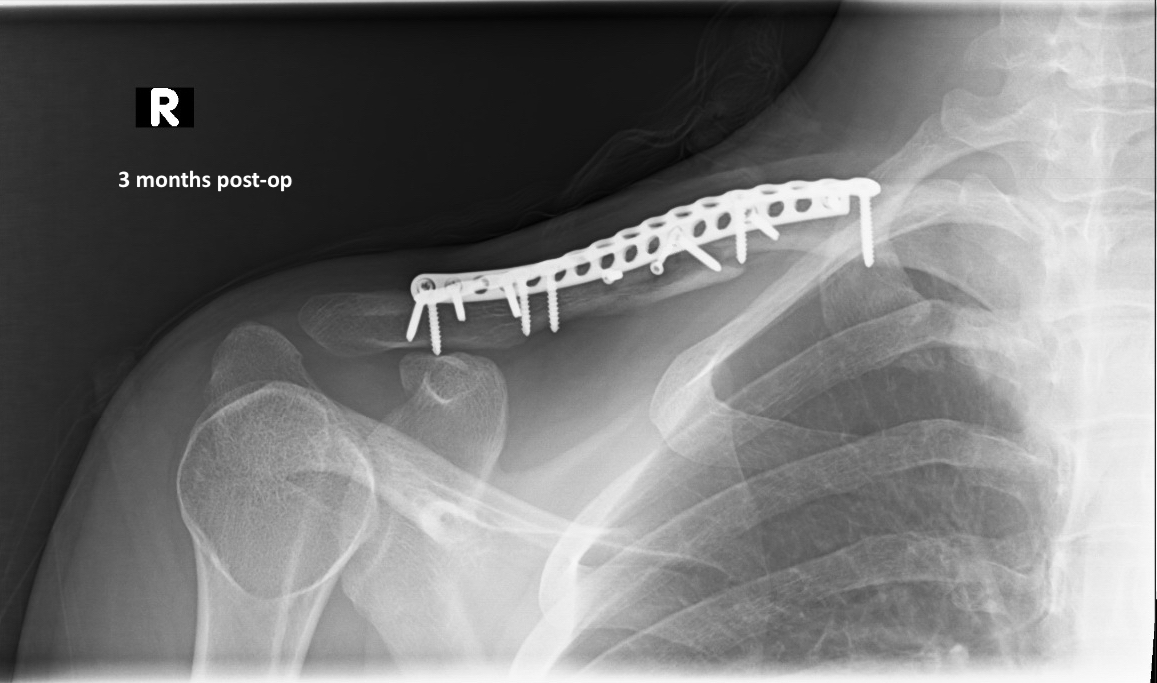

Supplement: Supplementary file 8 [file jetem-5-4-v6-supp8.jpeg]

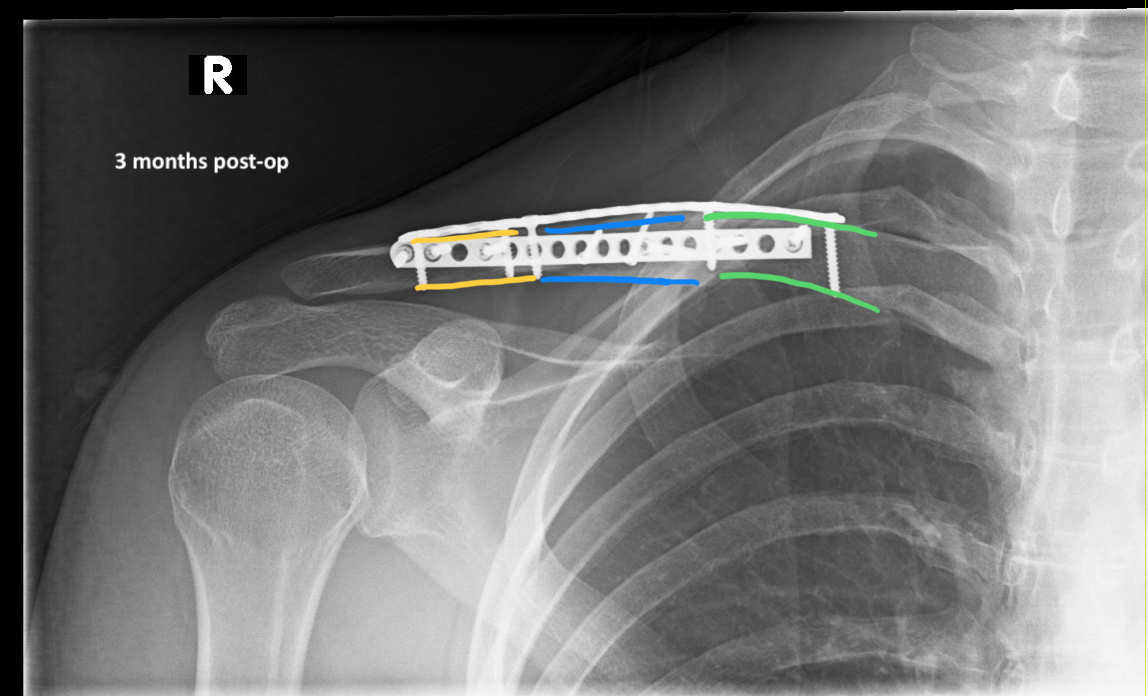

Supplement: Supplementary file 9 [file jetem-5-4-v6-supp9.jpeg]

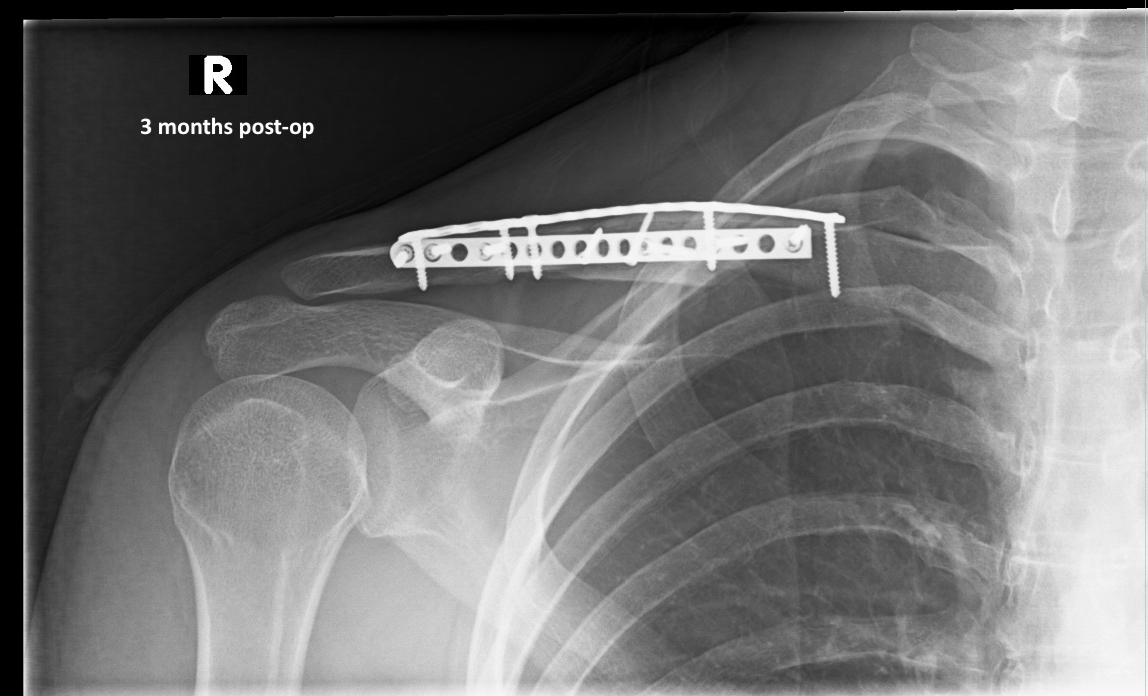

Supplement: Supplementary file 10 [file jetem-5-4-v6-supp10.jpeg]
